# Recent Advances in Cyclonucleosides: *C*-Cyclonucleosides and Spore Photoproducts in Damaged DNA

**DOI:** 10.3390/molecules171011630

**Published:** 2012-09-28

**Authors:** Yuichi Yoshimura, Hiroki Takahata

**Affiliations:** Faculty of Pharmaceutical Sciences, Tohoku Pharmaceutical University, 4-4-1 Komatsushima, Aoba-ku, Sendai 981-8558, Japan; Email: takahata@tohoku-pharm.ac.jp

**Keywords:** nucleosides, cyclonuclesides, *C*-cyclonucleosides, spore photoproduct, synthesis, damaged DNA, conformation, oxidative stress, UV, DNA repair

## Abstract

Cyclonucleosides which are fixed in a specific conformation around the glycosyl bond by a carbon and heteroatom chain constitute a unique category of nucleoside derivatives. Because they are structural analogs, cyclonucleosides and oligodeoxynucleotides containing them would be useful tools for investigating the biological functions and conformations of DNA, RNA as well as their steric interactions with proteins. *C*-Cyclonucleosides bridged by a carbon chain between the base and sugar moieties are the most attractive from the synthetic points of view as well as for use as biological tools. In this review, recent progress of the synthesis of *C*-cyclonucleosides is surveyed. Among the *C*-cyclonucleosides, 5′,8-*C*-cyclodeoxyadenosine is one of the well-known derivatives of which the first practical synthesis was reported over 30 years ago. Recently, 5′,8-*C*-cyclodeoxyadenosine has attracted considerable interest as a biomarker, since its formation in oxidatively-damaged DNA is considered to be related to various diseases and aging. Another important analogue of cyclonucleosides is a unique thymidine phosphate dimer, a so-called spore photoproduct, which has been found in photo-damaged DNA. Recent advances in the synthesis, mechanism-studies, and stereochemical preference of repairing enzymes related to 5′,8-*C*-cyclodeoxyadenosine and spore photoproducts are also reviewed.

## 1. Introduction

**Figure 1:**
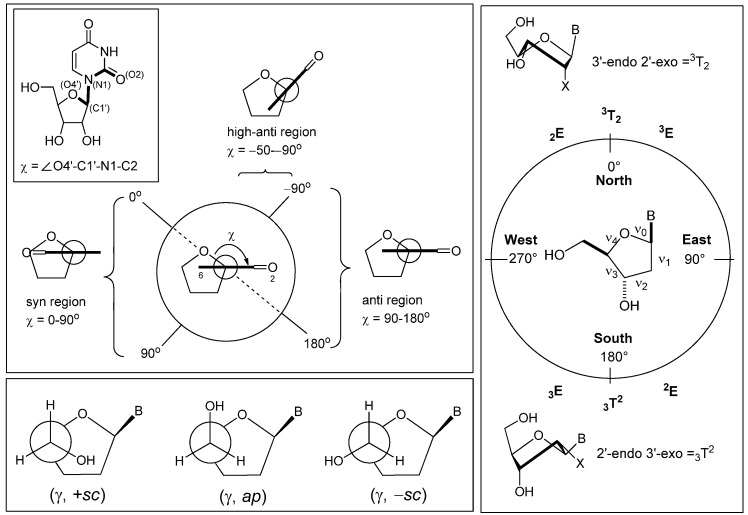
Conformations of nucleosides.

Nucleic acids contain genetic information and code blueprints for various proteins which are translated via mRNA. There is no doubt that nucleic acids are critical to life, since, once their systems are impeded, this is a fatal event in life itself in most cases. Indeed, based on this concept, many antitumor and antiviral drugs, acting on DNA, RNA and enzymes which utilize nucleic acids as a substrate, have been developed and are currently in use in clinical fields [1,2,3,4,5,6,7]. Similarly, nucleoside (nucleotide) mimics designed to interact with DNA (RNA) and to inhibit enzymes utilizing them are considered to be useful biological tools [8,9]. One such example would be a nucleoside analogue which is fixed in a specific conformation. The conformation of nucleosides is defined by three different parameters [10]: (1) conformation around the glycosyl bond (defined by the torsion angle χ), (2) conformation of the sugar portion (puckering, defined by a pseudorotational phase angle *P*; tan *P* = [(ν_4_ + ν_1_) − (ν_3_ + ν_0_)]/[2 ν_2_ (sin 36° + sin 72°)], (3) conformation around the C4′-5′ bond (defined by the torsion angle γ) (Figure 1). Conformational mimics of nucleosides corresponding to these three parameters have been prepared reported [11,12,13,14]. For example, it is well known that nucleoside derivatives, in which the sugar puckering is fixed in a *C*2′-endo (South) conformation are good conformational mimics of nucleotides found in an A-form duplex, e.g., RNA-RNA and RNA-DNA [11,12].

**Figure 2:**
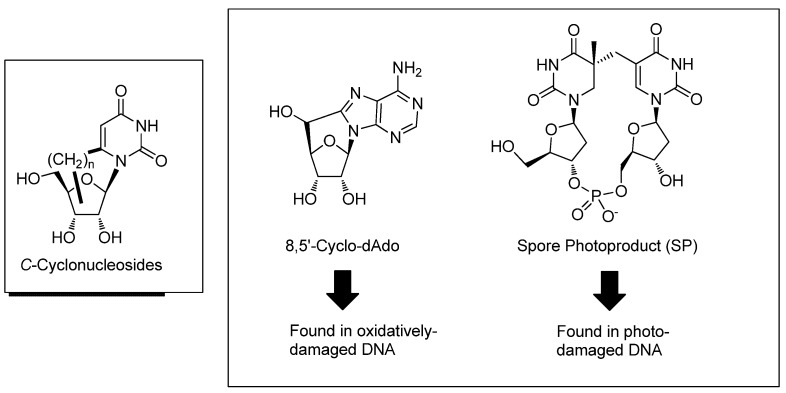
Structures of *C*-cyclonucleosides, 8,5′-*C*-cycloadenosine, and spore photoproduct.

On the other hand, cyclonucleosides that are fixed in a specific conformation around the glycosyl bond by a carbon and heteroatom chain also constitute a unique category of nucleoside derivatives [15]. Due to the structural bias existing in a molecule, cyclonucleosides and oligodeoxynucleotides containing them would be useful biological tools for investigating the functions and conformations of DNA, RNA as well as their steric interactions with proteins [15]. *C*-Cyclonucleosides are one of the classes of cyclonucleoside derivatives bridged by a carbon chain between the base and the sugar moieties and is attractive from the synthetic points of view as well as having potential for use as biological tools [15,16] (Figure 2). Since the construction of a bicyclo scaffold by which the *C*-cyclonucleoside is fixed in a different glycosyl torsion angle (*cf*, anti-, high-anti- and syn-orientation) would be a good synthetic target, various synthesis of *C*-cyclonucleoside derivatives have been reported [15,16]. In this review, recent progress in the synthesis of *C*-cyclonucleosides is surveyed.

Among the *C*-cyclonucleosides, 5′,8-*C*-cyclodeoxyadenosine is one of the well-known derivatives: its 5′-monophosphate derivative was originally found as a product of the reaction between adenosine 5′-monophosphate and hydroxyl radicals in 1968 [17] and the first practical synthesis of 5′,8-*C*-cyclodeoxyadenosine via the photoreaction of 5′-thiophenyladenosine was reported in 1976 [18]. 5′,8-*C*-Cyclodeoxyadenosine has recently attracted interest as a biomarker since its formation in oxidatively-damaged DNA is considered to be related to various diseases and aging [19,20,21,22,23,24].

Another important analogue of cyclonucleosides is a unique thymidine phosphate dimer, a so-called spore photoproduct (SP), which was found in photo-damaged bacterial spore DNA [25,26]. In the case of pyrimidine nucleotides, cyclobutane thymine dimers and (6-4) pyrimidine pyrimidone photoproducts are typical photoproducts [26]. Although it is likely that SP belongs to a thymine dimer category, its structure is different from other cyclobutane dimers. While SP was discovered over 40 years ago [27], the mechanism of its formation has remained unclear. In addition, SP-containing spore DNA is repaired by SP lyase, which is thought to be a component of the protection system of bacterial spores against photoreaction [25,26]. In SP, four stereoisomers at the C5 position of the thymine moiety can be formed; the stereochemical preferences at C5 position to be recognized by SP lyase is the point at issue (*vide infra*). Recent achievements in the synthesis, mechanism-studies, and the stereochmical preference of repairing enzymes concerning 5′,8-*C*-cyclodeoxyadenosine and spore photoproducts are also reviewed.

## 2. Synthesis of *C*-Cyclonucleosides

### 2.1. Classical Synthesis of C-Cyclonucleosides

**Scheme 1:**
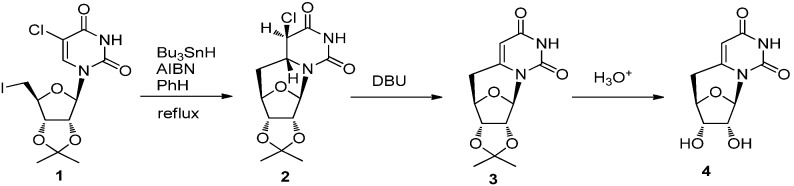
Synthesis of 5′,6-*C-*cyclouridine via radical cyclization.

In earlier synthesis of *C*-cyclonucleosides, radical cyclization reactions were commonly used to construct the carbon-carbon bridge between the base and sugar. A typical example of this is the synthesis of 5′,6-*C-*cyclouridine reported by Ueda and co-workers, in which the radical cyclization of 5-chloro-5′-iodouridine derivative **1** was employed as a key step [28]. The reaction gave 5,6-dihydrocyclouridine **2** stereoselectively and the double bond between 5- and 6-positions was generated by treatment with DBU. Deprotection of **3** gave 5′,6-*C-*cyclouridine [28] (Scheme 1).

**Scheme 2:**
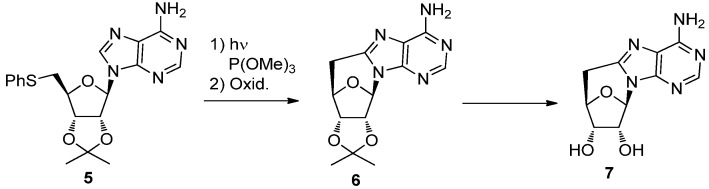
Synthesis of 5′,8-*C-*cycloadenosine via a photoreaction.

Similarly, Ueda *et al*. synthesized a 5′,8-*C-*cycloadenosine derivative by the radical cyclization of 5′-thiophenyladenosine **5** under photoreaction conditions [18]. The reaction coupled with oxidation gave 5′-deoxy-5′,8-*C-*cycloadenosine **7** after removal of the isopropylidene group [18] (Scheme 2).

**Scheme 3:**
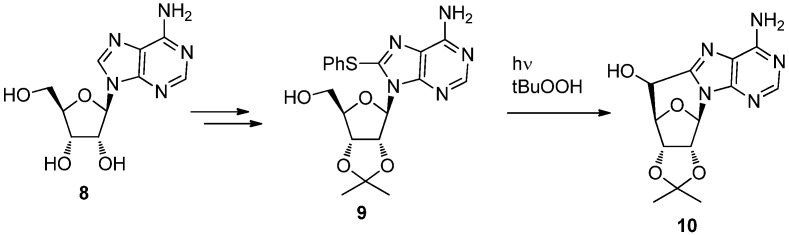
Synthesis of 5′,8-*C-*cycloadenosine via photoreaction of 8-thiophenoxyadenosine.

They also reported that the photoreaction of 8-thiophenoxyadenosine **9** obtained from adenosine **8** gave 5′,8-*C-*cycloadenosine **10** as a mixture of diastereomers at the 5′-position [29] (Scheme 3). When the reaction was initially reported, the mechanism of the formation of 5′,8-*C-*cycloadenosine **10** was unclear. Later, Chatgilialoglu and colleagues confirmed reaction mechanism during their studies of the reaction of the 8-bromoadenosine derivative, as well as additional chemical and biological aspects related to 5′,8-*C-*cycloadenosine (*vide infra*).

**Scheme 4:**
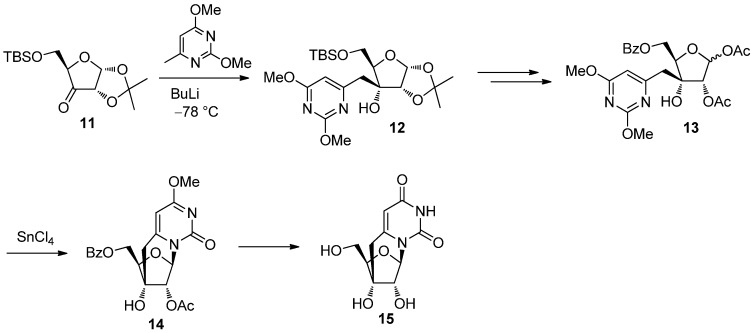
Synthesis of 3′,6-methanouridine via intramolecular glycosylation.

At the end of the 1980s and the early 1990s, we reported on an alternative synthesis of pyrimidine *C*-cyclonucleosides based on an intramolecular glycosylation reaction [30,31,32,33]. As shown in Scheme 4, 2,4-dimethoxy-6-methylpyrimidine was lithiated at the 6-methyl group and treated with the 3-ketosugar **11** to stereoselectively give a branched sugar derivative **12** [30]. The diacetate **13** derived from **12** was subjected to the intramolecular glycosylation mediated by SnCl_4_ to give the cyclonucleoside **14** from which 3′,6-methanouridine **15** was obtained [30] (Scheme 4).

By employing the radical cyclization and the intramolecular glycosylation reactions described above, various *C*-cyclonucleosides fixed in a certain conformation can be further prepared by connecting them to carbons of the sugar moiety [15,16,18,28,29,30,31,32,33].

### 2.2. Recent Advances in the Synthesis of C-Cyclonucleosides

**Scheme 5:**
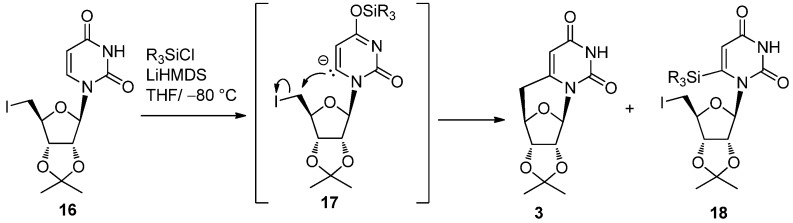
One step synthesis of 5′-deoxy-5′,6-*C*-cyclouridine using LiHMDS and a silylating agent.

As described above, radical cyclization is the most powerful tool for constructing a carbon-carbon bond between a base and sugar moieties in the synthesis of pyrimidine *C*-cyclonucleosides from uridine, since an alkyl radical generated on the sugar preferentially attacks the electron deficient 6-position of uracil. However, the method involves three reaction steps: (1) introduction of a chloro group at the 5-position, (2) radical cyclization, and (3) an elimination step to recover the 5,6-double bond. It was obvious that direct C-C bond formation between the 6- and 5′-positions would be the straightforward route to the preparation of 5′,6-*C*-cyclouridine. Although lithiation at the 6-position was reported [34], it was difficult to apply this in the synthesis of *C*-cyclouridine because the use of LDA was needed. We discovered that the use of LiHMDS, a rather much weaker base compared to LDA, in the presence of silylating agent permitted the 6-lithio derivative to be generated via a temporary 4-*O-*silylated derivative **17**, which spontaneously cyclized to give 5′-deoxy-5′,6-*C*-cyclouridine [35] (**3**). As shown in Scheme 5 and Table 1, both diphenylmethylsilyl chloride and diphenyldichlorosilane were effective in terms of producing 5′-deoxy-5′,6-*C*-cyclouridine (**3**) in excellent yields [35].

**Table 1:** Reaction yields of *C*-cyclouridine.

R_3_SiCl (equiv.)	Yield (%)	
	3	18
None	0	0
TMSCl (3.0)	56	42
Me(Ph)_2_SiCl (1.5)	83	trace
Ph_2_SiCl (3.0)	88	trace

**Scheme 6:**
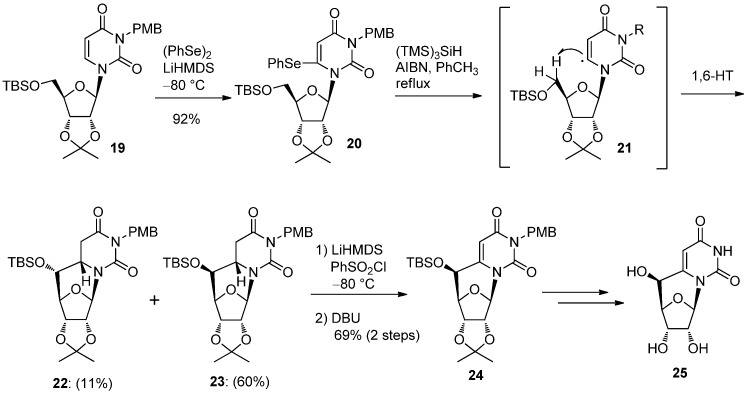
Synthesis of 5′,6-*C*-cyclouridine by a tandem radical 1,6-hydrogen transfer and cyclization reaction.

Our new synthetic concept in which carbon chain formation directed from 6 to 5′-position was further extended to 5′,6-*C*-cyclouridine **25** by developing a tandem radical 1,6-hydrogen transfer (1,6-HT) and a cyclization reaction [36], similar to that reported in the synthesis of purine *C*-cyclonucleoside (vide infra). The lithiation reaction mentioned above was used in preparing the 6-selenophenyl derivative **20** which, when treated with tris(trimethylsilyl)silane [(TMS)_3_SiH] and AIBN, gave a mixture of diastereomers **22** and **23** in 11 and 60% yields, respectively [36]. The reaction proceeds through an intermediate **21**, generated from **20**, in which 1,6-HT and the subsequent radical cyclization of the resulting 5′-radical occurred [36]. The recovery of the 5,6-double bond of the major (*S*)-isomer **23** gave the 5′,6-*C*-cyclouridine derivative **24** which could be converted to 5′,6-*C*-cyclouridine **25** [36] (Scheme 6).

**Scheme 7:**
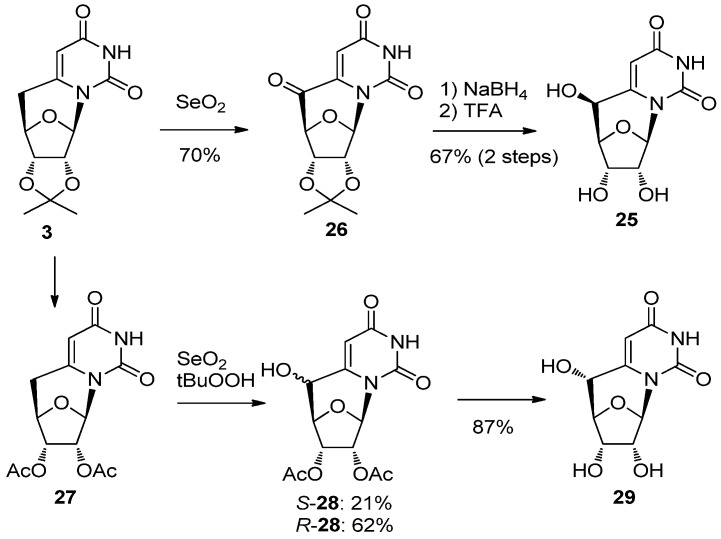
An alternative route for the synthesis of 5′-(*S*)-5′,6-*C*-cyclouridine (**25**) and 5′-(*R*)-5′,6-*C*-cyclouridine (**29**).

Very recently, MacLaughlin and co-workers reported an alternative route [37] for the synthesis of 5′-(*S*)-5′,6-*C*-cyclouridine (**25**) and 5′-(*R*)-5′,6-*C*-cyclouridine (**29**). Cyclonucleoside **3** was oxidized with SeO_2_ to give **26** which was reduced with NaBH_4_ followed by deprotection to furnish 5′-*S*-cyclouridine **25** exclusively [37]. Meanwhile the SeO_2_ oxidation of **27**, obtained from **3**, in the presence of tBuOOH predominantly gave *R*-**28**, which was converted to 5′-(*R*)-5′,6-*C*-cyclouridine **29** [37] (Scheme 7).

**Scheme 8:**
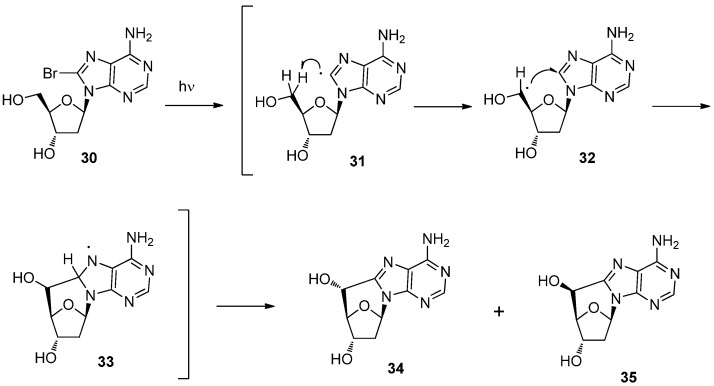
Mechanism of the conversion of 2′-deoxy-8-bromoadenosine (**30**) to 5′,8-cycloadenosines.

Before we reported on the tandem radical 1,6-HT and cyclization reactions of pyrimidine nucleosides, Chatgilialoglu and colleagues reported on a similar radical cascade reaction of 2′-deoxy-8-bromoadenosine (**30**) which gave the 5′,8-cycloadenosines **34** and **35** [38,39]. Based on their pioneering work, the reaction mechanism was shown to proceed as shown in Scheme 8 [38,40]. The photoreaction of 8-bromoadenosine **30** initiates the formation of a radical at the 8-position of the purine ring which gives rise to 1,6-HT to produce 5′-radical **32** [38,40]. The radical cyclization of **32** affords the intermediate **33**, the oxidation of which gives a mixture of 5′-(*R*)-cycloadenosine **34** and its 5′-(*S*)-isomer **35** [38,40] (Scheme 8).

**Scheme 9:**
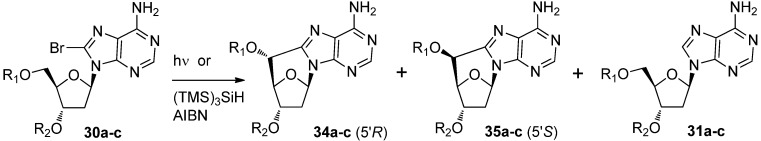
The effects of reagents, solvents, and protecting groups in the stereochemistry of radical cascade cyclization.

Regarding the radical cascade reaction to afford 5′,8-cycloadenosines, the effects of reagents, solvents, and protecting groups were investigated by Chatgilialoglu′s group [38,41] and a part of the results are summarized in Scheme 9 and the Table 2. The ratio of 5′-(*R*)-cycloadenosine **34** and 5′-(*S*)-isomer **35** in the reaction depends on the substrate used and the reaction conditions [38,41]. When the 5′-unprotected substrate was reacted in CH_3_CN, the ratio of **34**/**35** was approximately one [38,41]. In H_2_O or a CH_3_CN/H_2_O mixture, the formation of the (*R*)-epimer **34** was increased. Chatgilialoglu proposed that the pro-R conformer of 5′-radical, giving the (*R*)-epimer **34**, can be stabilized by a hydrogen bond in an aqueous solution [38,41]. On the other hand, when the substrate contains a bulky protecting group at the 5′-position, e.g., **31c**, the 5′-(*S*)-isomer **35b** is the major product, due to steric repulsion between the TBS group and adenine which favors the C5′-endo conformer: the TBSO group occupies a pseudoequtrial position in the newly formed 6-membered ring [41]. The conformations of the 5′-radicals were theoretically calculated using DFT B3LYP with 6-311++G(d,p) and the results were in good agreement with the experimental results [41] (Scheme 9).

**Table 2:** Summary of the effects of reagents, solvents, and protecting groups in the stereochemistry of radical cascade cyclization.

Comp.	Substituents	Conditions	Solvent	Relative yields (%)			34/35 ratio
				34	35	31	
**31a**	R_1_ = R_2_ = H	(TMS)_3_SiH	CH_3_CN	38	47	15	45/55
		h*ν*	H_2_O	31	7	ND	82/18
**31b**	R_1_ = H, R_2_ = TBS	(TMS)_3_SiH	CH_3_CN	32	38	30	45/55
		h*ν*	CH_3_CN/H_2_O	60	25	15	70/30
**31c**	R_1_ = R_2_ = TBS	(TMS)_3_SiH	CH_3_CN	8	70	22	10/90

**Scheme 10:**
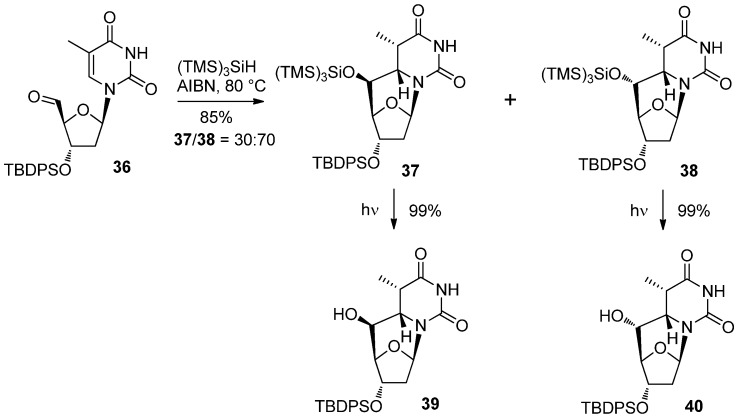
Radical cyclization of thymidine 5′-carbaldehyde **36** using (TMS)_3_SiH and photochemical deprotection.

The generation of 5′-radicals was not restricted to the use of the tandem 1,6-HT/cyclization cascade. More typically, treatment of the 5′-carbaldehyde with a suitable reducing agent can generate the 5′-radical, leading to the production of *C*-cyclonucleosides. Indeed, this type of radical cyclization had been reported over 20 years ago [42] and was considered to be one of the standards for synthesizing *C*-cyclonucleosides [43]. In most of the previous cases, Bu_3_SnH was used as a reducing agent under radical reaction conditions [42,43]. Chatgilialoglu’s group reported on a related radical cyclization using (TMS)_3_SiH [44]. The reaction of thymidine 5′-carbaldehyde **36** with (TMS)_3_SiH gave a mixture of 5′-(*S*)-*C*-cyclothymidines **37** and its 5′-(*R*)-isomer **38** in a ratio of 3:7 [44]. The result was quite interesting since reactions in which Bu_3_SnH was used afforded the 5′-(*S*)-isomers as the major products [45]. They also reported on the photochemical deprotection of tris(trimethylsilyl) group by which compounds **37** and **38** were converted to *C*-cyclothymidine derivatives **39** and **40** in excellent yields [44] (Scheme 10).

**Scheme 11:**
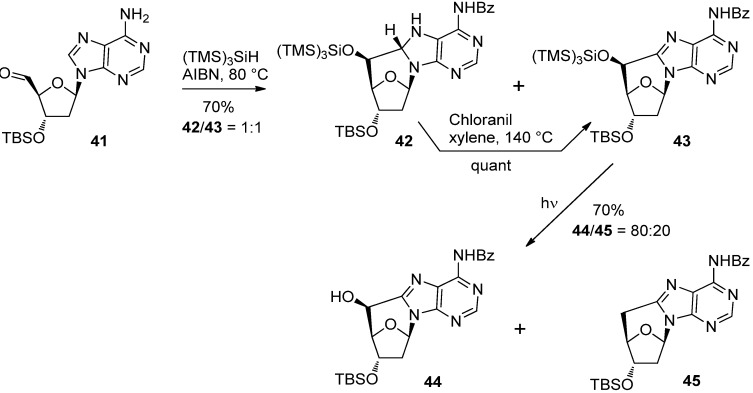
Radical cyclization of 2′-deoxyadenosine 5′-carbaldehyde **41** using (TMS)_3_SiH and photochemical deprotection.

The same reaction sequences were applied to 2′-deoxyadenosine 5′-carbaldehyde **41** [44]. In contrast to the case of thymidine, the radical cyclization of **41** gave 5′-(*S*)-*C*-cycloadenosine derivatives **42** and **43** as a single diastereomer at the 5′-position [44]. The former **42** could be converted into 5′-(*S*)-*C*-cycloadenosine **43** by oxidation with chloranil in refluxing xylene [44]. Photolysis of *C*-cycloadenosine **43**, however, gave a mixture of the desilylated **44** and 5′-deoxy derivative **45** [44] (Scheme 11).

**Scheme 12:**
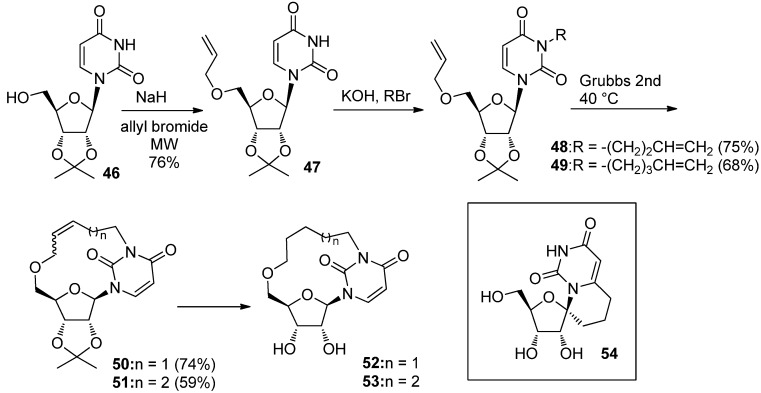
Synthesis of *C-*cyclonucleosides with fixed syn-conformations.

Compared to the synthesis of *C*-cyclonucleosides fixed in high-anti- and syn-forms, anti-fixed *C*-cyclonucleosides are rather easy to synthesize, since the synthesis of the corresponding branched nucleosides are needed prior to the construction of carbon-bridge in the case of syn-and high-anti conformational mimics. The recent and unique report by Len *et al.* showed that novel syn-fixed *C*-cyclonucleosides, bridged between N3 and 5′-O with 13- and 14-membered rings, were synthesized in short steps [46]. A sequence of selective *O-* and *N-*alkylations of the isopropylideneuridine **46** gave the dialkylated derivatives **48** and **49** [46]. Ring closing metathesis (RCM) is a powerful tool for the construction of various ring-size cycloalkene derivatives and was applied to the construction of 13- and 14-membered ring giving *C*-cyclouridine derivatives **50** and **51**, which were produced in satisfactory yields [46]. The resulting *C*-cyclouridines **52** and **53** were unique syn-fixed conformational analogues [46], the synthesis of which was quite limited to a few reports [47,48,49,50] including 1′,6-propanouridine [47,48] (**54**) (Scheme 12).

**Scheme 13:**
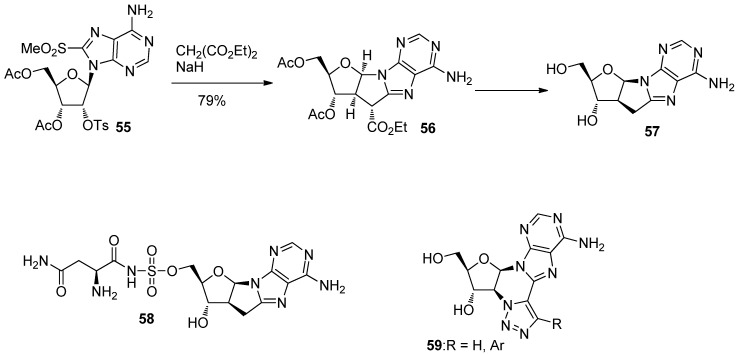
Synthesis of 2′,8-methanoadnosine (**57**) and its analogue **58**, a potential anti-parasitic agent.

The synthesis of a high-anti conformational analogue, 2′,8-methanoadenosine (**57**), on the other hand, was reported by Matsuda *et al*. in 1985 [51] and was achieved by a sequential reaction involving the addition/elimination of a malonate ion at the 8-position of adenine and intramolecular nucleophilic substitution at the 2′-position as shown in Scheme 13 [51]. In 2006, Sukuru *et al*. reported that 2′,8-methanoadenosine (**57**) was a potential inhibitor of the parasite asparaginyl-t-RNA synthase (AsnRS) from their docking simulation using the SLIDE software program [52]. In addition, a novel cycloadenosine derivative **58**, which they also synthesized, was revealed to have micromolar inhibitory activity against *Brugia malayi* AsnRS [52]. The work triggered the synthesis of **59**, an analogue of **57**, which had fluorescent properties and was expected to act as biological probes [53] (Scheme 13).

## 3. Formation of *C*-Cyclonucleosides in Damaged DNA

### 3.1. Purine C-Cyclonucleosides Found in Oxidatively-damaged Nucleic Acids

**Scheme 14:**
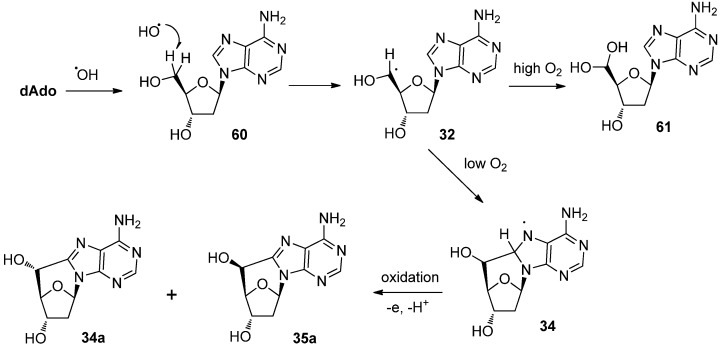
The reaction mechanism for the γ-radiolysis of 2′-deoxyadennosine giving *C*-cycloadenosine.

As described above, 2′-deoxy-5′,8-*C*-cycloadenosines **34a** and **35a** have been focused as major lesions in DNA because their formation is generally thought to be related to the oxidative-damage of DNA [38,54]. The first report of 5′,8-*C*-cycloadenosines appeared in 1968; a mixture of diastereomers of 5′,8-*C*-cycloadenosine 5′-monophosphate was reported to be formed by γ-irradiation of adenosine 5′-monophosphate [17]. The reaction mechanism, which involves γ-radiolysis to give the *C*-cycloadenosine is depicted in Scheme 14.

Hydroxyl radicals generated by the γ-irradiation of water attacks to C5′ of the deoxyadenosine **60** and a resulting H-abstraction gives rise to the C5′-radical **32** [54]. The subsequent radical cyclization to form a C5′-C8 bond produces the cyclonucleoside **33** which, on oxidation, gives the *C*-cycloadenosines **34a** and **35a** [54]. As described above, the (*R*)-isomer **34a** is the major product in the reaction of free deoxyadenosine in an aqueous solvent [38,41]. However, the ratio of (*R*)- and (*S*)-isomers depends on the reaction conditions, substrate, and solvents [38,41]. In addition, it was confirmed that molecular oxygen could suppress the formation of cyclonucleosides, leading to the formation of the hydrated 5′-aldehyde **61** in a model reaction [55]. The results clearly showed that a path exists, in which molecular oxygen attacks the 5′-radical, and competes with the radical cyclization [55] (Scheme 14). Similar results were observed in the case of γ-irradiated DNA: the formation of cyclodeoxyadenosine and -guanosine were observed under low oxygen conditions when calf thymus DNA was subjected to γ-irradiation, and the formation of cyclonucleosides was decreased with increasing oxygen concentration [56]. The (*R*)-isomers are also preferentially formed in γ-irradiated calf thymus DNA [56].

**Figure 3:**
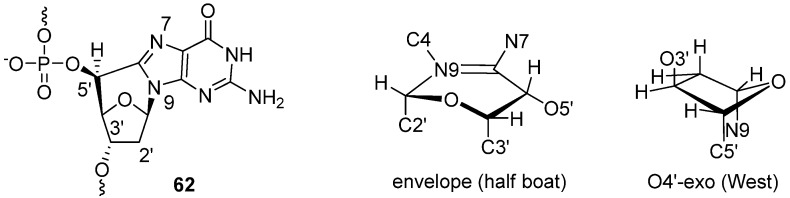
The structure of 5′,8-deoxyguanosine in DNA.

There is no doubt that DNA lesions, caused by the formation of structurally-rigid cyclonucleosides, perturb the structures of double-stranded DNA (dsDNA) and single-stranded DNA (ssDNA) [54,57]. The structural distortion of DNA contributed by 5′,8-cyclodeoxyadenosine affects various biological events: it blocks (1) the primer extension by T7 DNA polymerase and polymerization catalyzed by DNA pol δ [58], (2) chain elongation catalyzed by DNA pol η and exonuclease action by DNase III (TREX1) [59], and (3) binding of the TATA binding protein to the TATA box [60]. As in the case of 5′,8-cyclodeoxyadenosine, 5′,8-cyclodeoxyguanosine, which is also formed in oxidatively-damaged DNA, blocks DNA replication and induces mutations in *Escherichia coli* [61]. Therefore, efforts to understand the structure of DNA or oligodeoxynucleotide (ODN) in which cyclonucleosides are incorporated have been made [57,62]. Quite recently, Stone and his co-workers reported the structure of ODN duplex containing (*S*)-5′,8-*C*-cyclo-2′-deoxyguanosine **62**, as determined by molecular dynamics calculations and NMR analysis [63]. The cyclodeoxyguanosine **62** exhibited O4′-*exo *puckering (*P* = 280.2°) and the conformation of the six-membered ring including the carbon bridge of *C*-cyclo unit adopted an envelope (half boat) conformation, as shown in Figure 3 [63]. In spite of significant perturbations by cyclodeoxyguanosine **62**, Watson-Crick base pairing was conserved at the G-C pair of **62**. It also remains stacked to the neighboring T-A pair at the 3′-side of the cyclonucleoside. The structural distortion of the duplex by the introduction of **62** was more significant at the lesion site and the 5′-neighbor of the cycloG-C pair, where **62** perturbed the helical twist and base pair stacking. It is clear that these structural perturbations are associated with unusual O4′-*exo* puckering of **62** at the lesion site causes the thermodynamic destabilization of the duplex, as revealed by a 9 ºC decrease in the Tm value [63].

Cellular DNA constantly has chances to be damaged by various mechanisms including oxidative stress. Because it is difficult to completely avoid the formation of DNA lesions, the cell has several systems to protect DNA by repairing damaged lesions [64]. To repair thymidine photodimers, for example, direct reversing systems, e.g., photolyase, are involved (vide infra). In the case where direct reversals are impossible, repair systems that remove DNA lesions by excision of damaged base or nucleotide exist [64]. The former base excision repair (BER) system eliminates the damaged nucleobase by the action of DNA glycosylase and the resulting abasic site is recognized by AP endonuclease which cleaves the phosphodiester bond at the DNA lesion [64,65]. The latter nucleotide excision repair (NER) recognizes helix-distorting lesions and excludes lesion-caused nucleotides by hydrolyzing phosphodiseter bonds [64,65]. For repairing DNA lesions containing 5′,8-*C*-cycloadenosine, it is assumed that NER, and not BER, would be responsible from the following reasons: 1) *C*-cyclonucleosides are resistant to glycosyl bond cleavage due to their rigid structure [66], and 2) the purine base would remain in the DNA with a covalent bond connecting at the 5′-position of the lesion site, even if the enzymatic hydrolysis of glycosyl bond could occur. Indeed, the only reported repair system for DNA lesion containing purine 5′,8-*C*-cyclonucleoside is NER (*vide infra*). In an earlier work, a plasmid DNA containing either the 5′-(*R*)- or 5′-(*S*)-isomer of 2′-deoxy-5′,8-*C*-cycloadenosine was proved to be repaired by NER with a preference for the 5′-(*R*)-isomer containing lesion to the 5′-(*S*)-isomer, although neither the 5′-(*R*)- nor 5′-(*S*)-isomer are recognized by human DNA glycosylases active in BER [58]. Brooks *et al*. prepared DNA containing 5′-(*S*)-2′-deoxy-5′,8-*C*-cycloadenosine which was evaluated for DNA repair by mammalian cell extracts and living cells and the results showed that the DNA lesion was repaired by NER, but not BER [67]. Recent work by Jaruga *et al*. also revealed that NEIL1, a DNA glycosylase which is involved in the BER of oxidatively-damaged DNA, plays a role in the repair of both (*R*)- and (*S*)-isomers of 2′-deoxy-5′,8-*C*-cycloadenosine since the significant accumulation of these cyclonucleosides were observed in *neil1^-/-^* mice and concluded that NEIL1 is involved in NER, as well as acting as a DNA glycocylase in BER [68].

### 3.2. Formation of a Spore Photoproduct and Its Repair by Spore Photoproduct Lyase

**Scheme 15:**
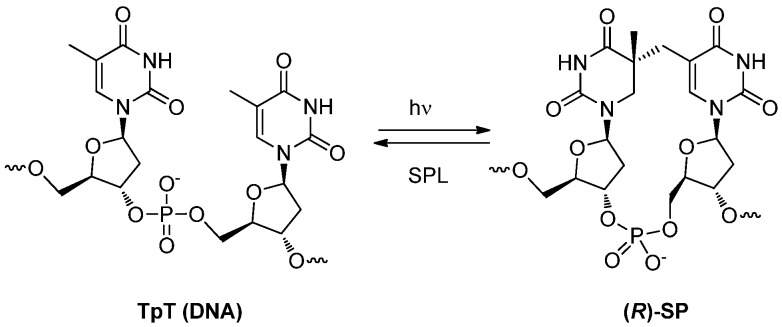
The formation of a spore photoproduct (SP).

DNA damage caused by UV radiation produces various photo-damaged nucleotides including thymine dimers [25,26]. Spore-forming bacteria, which cause many serious diseases in humans, are known to be extremely resistant to sterilization including oxidizing agents and UV or gamma irradiation [25]. A spore photoproduct (SP) belonging to a category of thymine dimers has been reported in photo-damaged bacterial spore DNA [27], the structure of SP, however, is different from the normal cyclobutane thymine dimer [25,26]. In addition the mechanism for its formation remains unclear. SP-containing spore DNA, on the other hand, is repaired by SP lyase (SPL) which constitutes a part of the protection system of bacterial spores from the UV-caused damage of DNA and which contributes to the extreme stability of spores against sterilization [25] (Scheme 15).

**Scheme 16:**
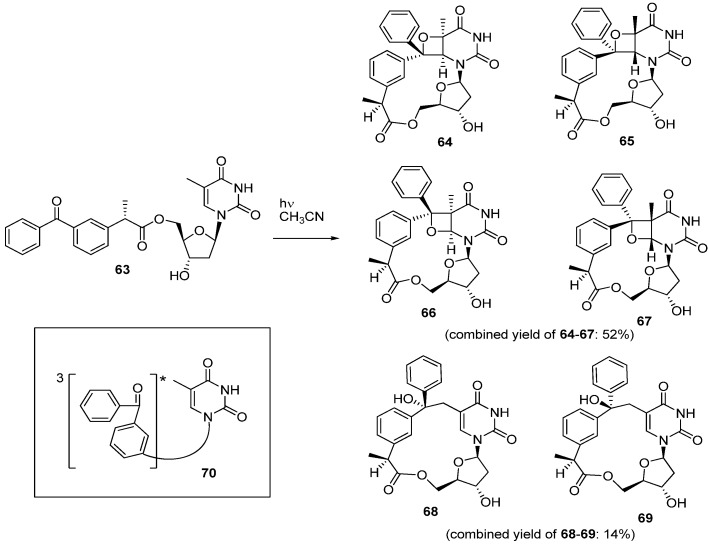
The photoreaction of 5′-benzophenone-thymidine dyad **63**.

To consider the reaction mechanism of SP as well as cyclobutane thymine dimer and (6-4) pyrimidine pyrimidone photoproducts, the study conducted by Miranda was interesting [69]. They synthesized a 5′-benzophenone-thymidine dyad **63** which was irradiated in acetonitrile through Pyrex, leading to formation of oxetane derivatives **64**–**67** (52%) and macrocyclic derivatives **68**–**69** (14%) [69]. In contrast, the photo-reaction of a similar 3′-benzophenone-thymidine dyad gave only a polymerized product [69]. The prevailing reaction mechanism for the formation of the oxetanes **64**–**67** is a Paternò-Büchi cycloaddition via the triplet excited state **70** [69]. Macrocyclic photoproducts **68–69**, on the other hand, arise from the same triplet **70** which abstracts a hydrogen of the methyl group of thymine followed by an intramolecular C-C bond formation of the resulting biradical intermediate (Scheme 16). The mechanism via triplet excited state **70** was supported by experimental evidence showing that an adiabatic photochemical cycloreversion of oxetane **64** gave rise to dyad **63** [69].

**Scheme 17:**
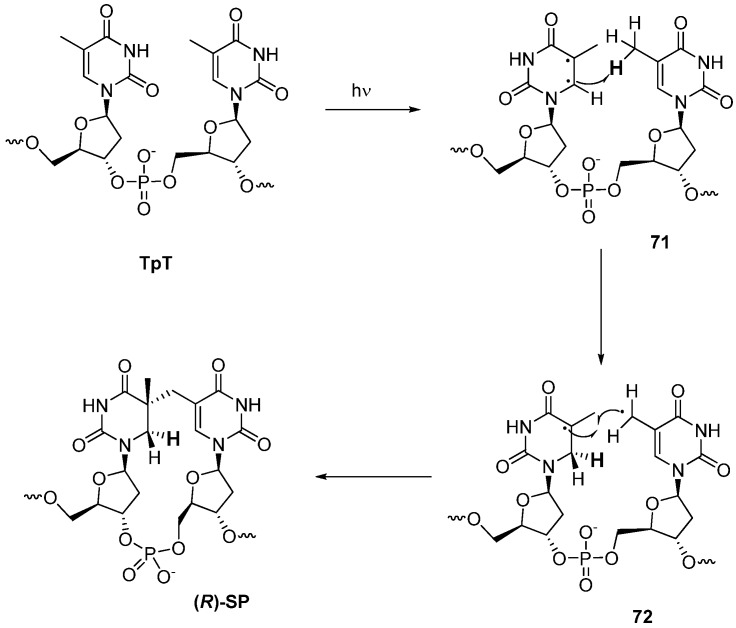
Mechanism for the formation of SP.

In 2010, Li *et al*. proposed a reaction mechanism for the formation of SP based on the structural elucidation of spore-photoproduct labeled with deuterium either at the 5-methyl hydrogen or the 6- and 5-methyl hydrogen of a dinucleotide of thymidine [70]. As depicted in Scheme 17, 5,6-biradicals **71** which are formed by the irradiation of the 5,6-double bond of 5′-thymidine abstracts a hydrogen from the methyl group of the 3′-thymidine to produce a pair of 5-α-thyminyl and 5,6-dihydrothymin-5-yl radicals **72** [70]. From the isotope effect observed in deuterium-incorporated derivative at the methyl group of 3′-thymidine, it was concluded that this step should be rate-limiting for the formation of SP [70]. Subsequent C-C bond formation between 5′-methyl and 5-yl radicals afforded the SP. This mechanism accounts for the recent results showing that SP is preferentially formed as an (*R*)-epimer at the 5-position of 5′-thymidine in spore DNA (also see below). Since both of the thymine moieties of **71** adopt an anti-conformation, this restricts the abstraction and transfer of hydrogen from the 3′-thymidine to the pro-S position of C6 in 5′-thymidine [70]. The resulting radicals react so rapidly that there is no chance for the relative positions of the substituents to change. As a result, the subsequent C-C bond formation occurs at the same side which the hydrogen has abstracted and produces (*R*)-SP predominantly [70] (Scheme 17). For the formation of (*S*)-SP, one thymine has to be converted from anti to syn-conformation around glycosidic bond. It is unlikely that such a conformational change occurs in dsDNA [25].

**Scheme 18:**
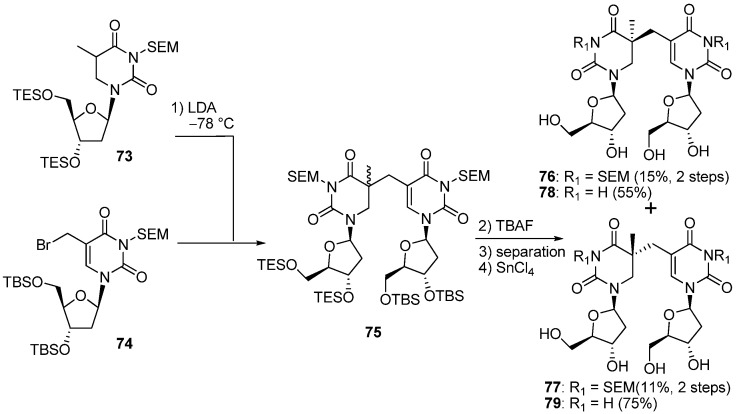
Synthesis of the SP nucleoside dimer.

**Scheme 19:**
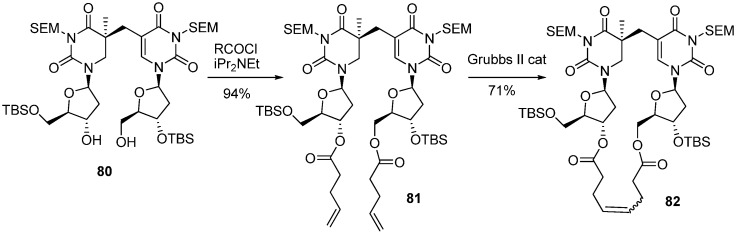
Synthesis of the cyclic SP nucleoside dimer.

As mentioned above, the spore-DNA lesion is efficiently repaired at the early germination phase by SPL, which is a DNA repair enzyme containing *S*-adenosylmethionine (SAM) and a special [4Fe-4S] cluster in its active site [71]. SPL catalyzes SP repair by a direct reversal reaction using a radical formed from the reductive cleavage of SAM in the active site [71]. However, the mechanism of the catalytic reaction by SPL is unclear. One important question in SPL is which C5-stereoisomer is successfully recognized and reversed to the normal thymidine dinucleotide. In principal, both 5-(*R*)- and 5-(*S*)-isomers of SP can be formed by the UV-irradiation of spore DNA. Regarding the stereochemistry of SP, the natural lesion might be formed as the 5-(*R*)-epimer, since the DNA in spores adopts an A-like conformation by binding to the acid-soluble protein (SASP) [72]. The steric constrains of the A-form duplex are considered to favor the formation of 5-(*R*)-SP, as discussed above. On the other hand, it has been reported that spore DNA exists exclusively as the B-form [73], thus, the possibility that a 5-(*S*)-isomer of SP could be formed cannot be completely ruled out. To obtain an answer to the question of the stereochemistry regarding SP recognition, model compounds that could be recognized by SPL were synthesized. Carell and co-workers reported the synthesis of both stereoisomers of the SP nucleoside dimer [74]. Based on the first synthesis of SP reported by Begley [75], 5,6-Dihydrothymidine **73** was lithiated at the 5-position by LDA, then treated with an allylbromide derivative **74** to give a mixture of diastereoisomers **75** [74]. Subsequent deprotection by TBAF followed by separation by reverse-phased HPLC gave the SEM-protected SP nucleoside dimers **76** and **77** in 15% and 11% respectively. Finally the SEM group was removed by treatment with SnCl_4_ to afford the SP nucleosides **78** and **79** [74] (Scheme 18). It was difficult to assign the stereochemistry at C5 of **78** and **79** in which a carbon-bridge was constructed due to the high flexibility of the molecules. To solve this problem, Carell *et al*. synthesized macrolactones **82** in which structural rigidity was introduced, and their structures were elucidated.^74^ The SEM-protected SP nucleoside dimer **80** which was obtained by the same methods described above was treated with 4-pentenoyl chloride to give a diester **81** [74]. Macrocyclization of **81** was achieved by the RCM reaction using the Grubbs II catalyst to give a rigid SP nucleoside dimer **82** in 71% yield (*E* : *Z* = 95 : 5) [74] (Scheme 19).

The stereochemistry at C5 of **82** was confirmed to have an *S* configuration from NOESY experiments [74]. By transferring the results for **78** and **79**, the structures of **78** and **79** were determined to be the C5-*S*- and C5-*R*-isomers respectively [74]. The SP nucleoside dimer **78** and **79** were used to study the enzymatic reaction of SPL isolated from *Geobacillus stearothermophilus*. The study showed that only the *S-*isomer **78** could be recognized and repaired by SPL and was consistent with their previous results obtained using SPL from *Bacillus subtilis* [76].

**Scheme 20:**
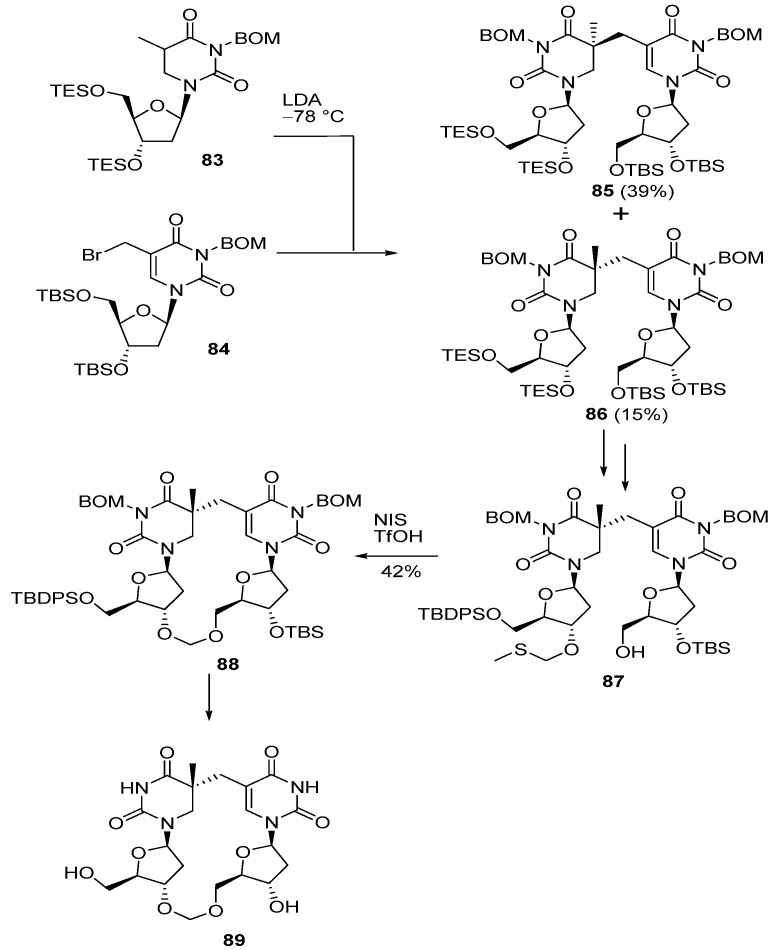
Synthesis of a methylene mimic of SP.

**Scheme 21:**
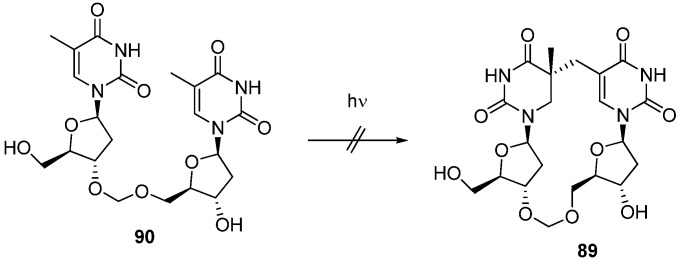
Attempt to obtain the SP analogue **89** by the photoreaction of **90**.

In contrast, Bardet and co-workers pursued NMR studies and DFT calculations of SP isolated and unambiguously determined the absolute configuration of the C5 of SP as *R* [77]. Broderick *et al*. also reported the synthesis of SP in which the stereochemical assignments at C5 were unambiguously confirmed by NOESY and ROESY [78]. An enzymatic assay using stereochemically defined synthetic SP demonstrated that the SPL from *Clostridium acetobutylicum* repairs (*R*)-SP specifically [78]. In addition, Li and his colleagues reported results supporting *R*-preference of SPL [79]. As Carell reported a formacetal (methylene) derivative of cyclobutane thymine dimer [80], they designed an SP analogue in which a methylene linker was introduced in place of the phosphodiester linkage [79]. The synthesis of the protected SP nucleoside dimer **85** and **86** was achieved by a method similar to that described above. After manipulation of the protecting group, the 3′-methylthiomethyl derivative **87** was prepared and was subjected to linker forming reaction by treatment with NIS and TfOH followed by deprotection to give the 5*R*-methylene SP isostere **89** [79] (Scheme 20). Li *et al*. also attempted a photoreaction of the methylene analogue of TpT **90**. However, UV irradiation of **90** did not give the desired SP analogue **89** under established photochemical procedures used to produce SP [79] (Scheme 21). The structure of the 5*R*-methylene SP analogue **89** was determined by X-ray analysis and ROESY spectroscopy, as well as DFT calculations (optimized at B3LYP/6-31+G(d,p) level), and proved to have similar structure to SP [79]. An enzymatic investigation using **89** revealed that the 5*R*-methylene SP analogue could be a substrate for SPL [79]. The failure to prepare the methylene SP analogue **89** by the photoreaction of 90 suggests that natural TpT adopts a different conformation with the methylene analogue **90** which lacks the negative charge of the phosphodiester bond [79]. Compared with this result, it is noteworthy that the 5*R*-methylene SP analogue **89** was converted into **90** by the action of SPL with a slightly decreased reaction rate compared to that for the natural substrate 5*R*-SP nucleotide dimer [79]. The result also suggests that SPL dominantly recognizes two thymine residues fixed by the phosphodiester linkage and that the methylene bridge functions to hold these thymine residues in the correct positions, similar to the function of the phosphodiester linkage of SP [79]. 

**Figure 4:**
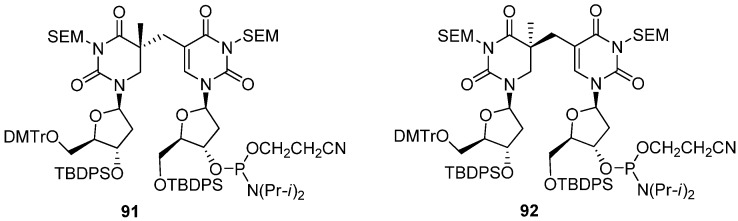
The structures of SP-amidite blocks **91** and **92** to prepare oligodeoxynucleotide.

Although conflicting results were reported by Carell and Li, it is likely that SPL specifically recognizes (*R*)-SP as a substrate. Quite recently, Carell *et al*. also reported that SPL efficiently repairs oligonucleotides containing (*R*)-SP, not (*S*)-SP [81]. In this study, they determined the stereochemistries at C5 by X-ray crystallographic analysis of SP-containing oligodepoxynucleotides, which were prepared using SP-amidite **91** and **92**, complexed with DNA polymerase I from *Geobacillus stearothermophilus* [81] (Figure 4). The absolute stereoconfiguration, obtained by X-ray analysis, was in agreement with findings reported by Broderick [78]. The 5*R*-SP lesion induced only minor structural distortions and was able to form a duplex with almost perfect Watson-Crick base pairing [81]. It is noteworthy that the missing phosphodiester backbone did not distort the double helix structure [81]. In contrast, the 5*S*-SP lesion disturbed the duplex structure significantly and it was difficult to fit a phosphodiester linkage into the missing part [81]. The result was in good agreement with idea that the structural constraints in the B-form duplex impede the formation of 5*S*-Sp lesions [81].

Quite recently, the first crystal structure of SPL in complex with [4Fe-4S] cluster and SAM in the absence and presence of an (*R*)-SP DNA lesion has been reported [82]. This epoch-making report provides high resolution structures of the active-site of SPL which afford fundamental insights regarding the DNA lesion recognition and the catalytic mechanism of SPL. (*R*)-SP, which is flipped out of bound DNA into the active site of SPL by the assistance of β-hairpin close to the binding pocket, mostly interacts with SPL at the base moieties [82]. Since the lack of phosphate linkage does not seem to affect the orientation of the nucleobase moieties in the active site of SPL, this successfully accounts for the results by Carell [81] described above and the preferential recognition of (*R*)-SP, not (*S*)-SP. Additionally, the results clearly revealed the reaction mechanism of SPL: an electron-transfer from the [4Fe-4S] cluster to SAM occurs after binding of (*R*)-SP. The resulting 5’-radical of 5’-deoxyadenosine abstracts pro-*R* hydrogen from the C6 of the 5’-dihydrothymine moiety. This triggers to form the repaired 5’-thymine residue and a 3’-thymine allylic radical which takes a hydrogen from cysteine to conclude the repair cycle [82]. 

## 4. Conclusions

*C*-Cyclonucleosides are unique nucleoside analogues which are fixed in a specific conformation around the glycosyl bond by a carbon chain. Since the construction of a bicyclo scaffold fixed in a different glycosyl torsion angle is a good synthetic target, the synthesis of a wide variety of *C*-cyclonucleoside derivatives have been reported to date. It is obvious that these synthetic efforts largely contributed to the progress made in nucleoside chemistry. The structural bias existing in *C*-cyclonucleosides permits them to be used a conformational probes in investigating the functions and conformations of nucleosides, nucleotides, DNA, RNA as well as their steric interactions with proteins. The interactions of 5′,8-cycloadenosine and its 5′-monophosphate with several enzymes including adenylate kinase and adenosine deaminase were investigated prior to the 1980s [83,84]. Enzymatic studies of ribonuclase A (RNase A) using 5′,6-*C*-cyclouridine 2′,3′-cyclicphosphate and other *C*-cyclouridine derivatives revealed that RNase A preferentially recognizes an anti-conformation [85]. While the focus of *C*-cyclonucleosides was on their use as biological tools, 5′,8-*C*-cyclodeoxyadenosine has attracted considerable attention as a biomarker, since its formation in oxidatively-damaged DNA is considered to be related to various diseases and aging, as described above. Thus, studies of the conformation of DNA lesions containing a 5′,8-*C*-cyclodeoxyadenosine unit are important in terms of understanding how conformational perturbation influences the function of genes and the transfer of genetic information. This is also the case for the formation and repair of SP lesions. SP has a unique macrocyclic structure connected by an unusual C-C bond between two thymines and a phosphodiester linkage. The formation of SP is related to the photo-damage of DNA and the repair system of SP is highly interesting as a DNA protection strategy. SPL corresponds to the repair of DNA lesions and catalyzes the direct reversion to thymine moieties. It is noteworthy that the specific recognition of 5*R*-SP by SPL has been clarified by investigations in which *C*-cyclonucleoside derivatives played a major role.
